# 

**Published:** 1888-04

**Authors:** 


					﻿NOTICE.
All articles for publication, books for review, business communications, etc.,
must be sent to Editors, 1123 Spruce Street, Philadelphia.
Subscribers will please notice that this Journal will be sent until arrears
are paid and it is ordered discontinued.
The best is always the cheapest; but the cheapest is not
always the best, Just here THE HOMOEOPATHIC PHY-
SICIAN contradicts dll previous experience in being both
the cheapest and the best. See that your friends know this ! !
WAITED.
The following back numbers of The Homceopathic Physician are wanted;
twenty-five cents will be paid for each number received in good condition.
Please look over your odd journals and see if you can help us. Write your
name om the wrapper. |
Address The Homceopathic Physician,
1123 Spruce Street, Philadelphia.
Volume First: Febuary, March, April, May, and June.
Volume Third: October.
				

